# (*SP*-4-2)-Chlorido{*N*-[2-(diphenyl­phosphan­yl)benzyl­idene]benzyl­amine-κ^2^ *P*,*N*}(meth­yl)palladium(II)

**DOI:** 10.1107/S1600536811040074

**Published:** 2011-10-05

**Authors:** Haleden Chiririwa, Reinout Meijboom

**Affiliations:** a Research Centre for Synthesis and Catalysis, Department of Chemistry, University of Johannesburg, PO Box 524 Auckland Park, Johannesburg 2006, South Africa

## Abstract

In the title Pd^II^ complex, [Pd(CH_3_)Cl(C_26_H_22_NP)], the Pd^II^ atom is coordinated in a slightly distorted square-planar geometry by the imino N and phosphane P atoms of the ligand, by one chloride ion and by a methyl ligand. The methyl group is *trans* to the N atom of the ligand.

## Related literature

For structures with related ligands, see: Coleman *et al.* (2001 ▶); Ghilardi *et al.* (1992 ▶); Sanchez *et al.* (1998 ▶, 1999 ▶, 2001 ▶); Chiririwa *et al.* (2011 ▶). 
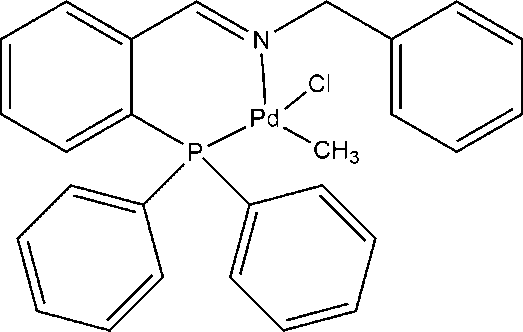


## Experimental

### 

#### Crystal data

[Pd(CH_3_)Cl(C_26_H_22_NP)]
*M* *_r_* = 536.30
Monoclinic, 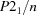

*a* = 10.0147 (8) Å
*b* = 21.8935 (18) Å
*c* = 10.7478 (8) Å
β = 94.192 (2)°
*V* = 2350.2 (3) Å^3^
*Z* = 4
Mo *K*α radiation
μ = 0.99 mm^−1^
*T* = 173 K
0.13 × 0.12 × 0.03 mm

#### Data collection

Bruker SMART APEX diffractometer
Absorption correction: multi-scan (*SADABS*; Sheldrick, 1997 ▶) *T*_min_ = 0.883, *T*_max_ = 0.971
32545 measured reflections
5809 independent reflections
4857 reflections with *I* > 2σ(*I*)
*R*_int_ = 0.043

#### Refinement

*R*[*F* ^2^ > 2σ(*F* ^2^)] = 0.034
*wR*(*F* ^2^) = 0.091
*S* = 1.03
5809 reflections
281 parameters
H-atom parameters constrained
Δρ_max_ = 1.21 e Å^−3^
Δρ_min_ = −0.62 e Å^−3^

### 

Data collection: *APEX2* (Bruker, 2007 ▶); cell refinement: *APEX2* and *SAINT-Plus* (Bruker, 2007 ▶); data reduction: *SAINT-Plus* and *XPREP* (Bruker, 2007 ▶); program(s) used to solve structure: *SHELXS97* (Sheldrick, 2008 ▶); program(s) used to refine structure: *SHELXL97* (Sheldrick, 2008 ▶); molecular graphics: *X-SEED* (Barbour, 2001 ▶); software used to prepare material for publication: *SHELXL97*.

## Supplementary Material

Crystal structure: contains datablock(s) I, global. DOI: 10.1107/S1600536811040074/im2317sup1.cif

Structure factors: contains datablock(s) I. DOI: 10.1107/S1600536811040074/im2317Isup2.hkl

Additional supplementary materials:  crystallographic information; 3D view; checkCIF report

## Figures and Tables

**Fig. 1.**
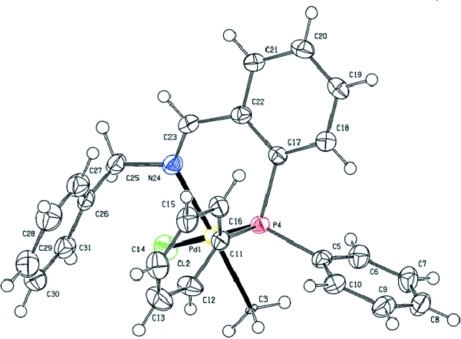
The structure of the title compound, showing 50% probability displacement ellipsoids.

## supplementary crystallographic information

### Comment

In recent years, palladium complexes with iminophosphane ligands of the type *N*-[(2-diphenylphosphanyl)benzylidene]amine type have been used as catalyst precursors for a range of organic reactions. Our group is interested in these types of complexes and we recently reported one such type of complex (Chiririwa *et al.,* 2011). The molecular structure of the title compound revealed a slightly distorted square planar geometry around the palladium metal center. The Pd—P distance of 2.1939 (7) Å is within the expected range and close to the values determined for the dihalide complexes of the same ligand (2.1925 (9) Å, Coleman *et al.,* 2001).

### Experimental

To a solution of the precursor [PdClMe(COD)] (0.07 g, 0.27 mmol) in anhydrous CH_2_Cl_2_ (10 ml) was added the calculated amount of iminophosphane ligand in CH_2_Cl_2_ solution, and the reaction wmixture as stirred at room temperature for 1 h. The yellow solution was then concentrated under reduced pressure to half volume and the addition of hexane caused precipitation of complex, which was filtered off, washed with Et_2_O and dried under vacuum for 4 h. Orange crystals of the title compound were obtained in 50% yield. Crystals suitable for X-ray diffraction studies were obtained by slow evaporation of a DMSO-d_6_/CH_2_Cl_2_ solution of the title compound at room temperature.

### Refinement

The aromatic, methylene, and methyl H atoms were placed in geometrically idealized positions (C—H = 0.95–0.98) and constrained to ride on their parent atoms with *U*_iso_(H) = 1.2*U*_eq_(C) for aromatic and methylene H atoms, and *U*_iso_(H) = 1.5*U*_eq_(C) for methyl H atoms respectively.

### Crystal data

**Table:**

[Pd(CH_3_)Cl(C_26_H_22_NP)]	*F*(000) = 1088
*M**_r_* = 536.30	*D*_x_ = 1.516 Mg m^−^^3^
Monoclinic, *P*2_1_/*n*	Mo *K*α radiation, λ = 0.71073 Å
Hall symbol: -P 2yn	Cell parameters from 7032 reflections
*a* = 10.0147 (8) Å	θ = 1.9–30.7°
*b* = 21.8935 (18) Å	µ = 0.99 mm^−^^1^
*c* = 10.7478 (8) Å	*T* = 173 K
β = 94.192 (2)°	Needle, orange
*V* = 2350.2 (3) Å^3^	0.13 × 0.12 × 0.03 mm
*Z* = 4	

### Data collection

**Table:**

Bruker SMART APEX diffractometer	5809 independent reflections
Radiation source: fine-focus sealed tube	4857 reflections with *I* > 2σ(*I*)
graphite	*R*_int_ = 0.043
Detector resolution: 0 pixels mm^-1^	θ_max_ = 28.3°, θ_min_ = 2.1°
n/a scans	*h* = −13→13
Absorption correction: multi-scan (*SADABS*; Sheldrick, 1997)	*k* = −29→29
*T*_min_ = 0.883, *T*_max_ = 0.971	*l* = −14→13
32545 measured reflections	

### Refinement

**Table:**

Refinement on *F*^2^	Primary atom site location: structure-invariant direct methods
Least-squares matrix: full	Secondary atom site location: difference Fourier map
*R*[*F*^2^ > 2σ(*F*^2^)] = 0.034	Hydrogen site location: inferred from neighbouring sites
*wR*(*F*^2^) = 0.091	H-atom parameters constrained
*S* = 1.03	*w* = 1/[σ^2^(*F*_o_^2^) + (0.0424*P*)^2^ + 2.9231*P*] where *P* = (*F*_o_^2^ + 2*F*_c_^2^)/3
5809 reflections	(Δ/σ)_max_ < 0.001
281 parameters	Δρ_max_ = 1.21 e Å^−^^3^
0 restraints	Δρ_min_ = −0.62 e Å^−^^3^

### Special details

**Table:**

Geometry. All e.s.d.'s (except the e.s.d. in the dihedral angle between two l.s. planes) are estimated using the full covariance matrix. The cell e.s.d.'s are taken into account individually in the estimation of e.s.d.'s in distances, angles and torsion angles; correlations between e.s.d.'s in cell parameters are only used when they are defined by crystal symmetry. An approximate (isotropic) treatment of cell e.s.d.'s is used for estimating e.s.d.'s involving l.s. planes.
Refinement. Refinement of *F*^2^ against ALL reflections. The weighted *R*-factor *wR* and goodness of fit *S* are based on *F*^2^, conventional *R*-factors *R* are based on *F*, with *F* set to zero for negative *F*^2^. The threshold expression of *F*^2^ > σ(*F*^2^) is used only for calculating *R*-factors(gt) *etc*. and is not relevant to the choice of reflections for refinement. *R*-factors based on *F*^2^ are statistically about twice as large as those based on *F*, and *R*- factors based on ALL data will be even larger.

### Fractional atomic coordinates and isotropic or equivalent isotropic displacement parameters (Å^2^)

**Table:**

	*x*	*y*	*z*	*U*_iso_*/*U*_eq_	
Pd1	0.14680 (2)	0.803769 (10)	0.037830 (19)	0.02303 (7)	
Cl2	0.00399 (7)	0.71581 (4)	0.04518 (7)	0.03620 (17)	
C3	0.03775 (19)	0.84358 (9)	0.17933 (18)	0.0076 (4)	
H3A	0.0318	0.8878	0.1664	0.011*	
H3B	0.0837	0.8351	0.2612	0.011*	
H3C	−0.0526	0.8261	0.1757	0.011*	
P4	0.27908 (6)	0.88365 (3)	0.03775 (6)	0.02086 (14)	
C5	0.2990 (3)	0.93431 (13)	0.1722 (2)	0.0244 (5)	
C6	0.3269 (3)	0.90852 (15)	0.2906 (3)	0.0320 (6)	
H6	0.3298	0.8654	0.2999	0.038*	
C7	0.3503 (3)	0.94591 (17)	0.3946 (3)	0.0378 (7)	
H7	0.3684	0.9283	0.4749	0.045*	
C8	0.3470 (3)	1.00886 (17)	0.3808 (3)	0.0383 (7)	
H8	0.3635	1.0344	0.4517	0.046*	
C9	0.3199 (3)	1.03468 (15)	0.2641 (3)	0.0359 (7)	
H9	0.3178	1.0778	0.2552	0.043*	
C10	0.2958 (3)	0.99750 (14)	0.1599 (3)	0.0289 (6)	
H10	0.2770	1.0154	0.0800	0.035*	
C11	0.2327 (3)	0.93237 (12)	−0.0962 (2)	0.0230 (5)	
C12	0.0979 (3)	0.94571 (15)	−0.1249 (3)	0.0342 (7)	
H12	0.0322	0.9300	−0.0741	0.041*	
C13	0.0595 (3)	0.98184 (17)	−0.2272 (3)	0.0432 (8)	
H13	−0.0324	0.9912	−0.2457	0.052*	
C14	0.1539 (3)	1.00428 (16)	−0.3024 (3)	0.0399 (8)	
H14	0.1270	1.0289	−0.3726	0.048*	
C15	0.2879 (3)	0.99095 (15)	−0.2756 (3)	0.0354 (7)	
H15	0.3528	1.0063	−0.3277	0.042*	
C16	0.3278 (3)	0.95514 (14)	−0.1728 (3)	0.0282 (6)	
H16	0.4200	0.9461	−0.1546	0.034*	
C17	0.4509 (3)	0.86061 (13)	0.0161 (2)	0.0226 (5)	
C18	0.5588 (3)	0.89176 (13)	0.0761 (3)	0.0261 (6)	
H18	0.5423	0.9237	0.1330	0.031*	
C19	0.6903 (3)	0.87685 (14)	0.0541 (3)	0.0292 (6)	
H19	0.7625	0.8981	0.0968	0.035*	
C20	0.7159 (3)	0.83128 (15)	−0.0296 (3)	0.0308 (6)	
H20	0.8055	0.8209	−0.0444	0.037*	
C21	0.6097 (3)	0.80071 (14)	−0.0920 (3)	0.0297 (6)	
H21	0.6273	0.7703	−0.1517	0.036*	
C22	0.4772 (3)	0.81369 (13)	−0.0687 (3)	0.0250 (6)	
C23	0.3733 (3)	0.77818 (14)	−0.1409 (3)	0.0281 (6)	
H23	0.3998	0.7590	−0.2144	0.034*	
N24	0.2515 (2)	0.77023 (11)	−0.1160 (2)	0.0268 (5)	
C25	0.1677 (3)	0.73599 (14)	−0.2122 (3)	0.0320 (6)	
H25A	0.1118	0.7058	−0.1712	0.038*	
H25B	0.2261	0.7135	−0.2668	0.038*	
C26	0.0787 (3)	0.77936 (14)	−0.2899 (3)	0.0298 (6)	
C27	0.1329 (3)	0.81835 (16)	−0.3762 (3)	0.0382 (7)	
H27	0.2268	0.8186	−0.3838	0.046*	
C28	0.0509 (4)	0.85650 (18)	−0.4506 (3)	0.0459 (8)	
H28	0.0885	0.8829	−0.5089	0.055*	
C29	−0.0870 (4)	0.85608 (18)	−0.4398 (3)	0.0471 (8)	
H29	−0.1435	0.8819	−0.4916	0.057*	
C30	−0.1421 (3)	0.81823 (17)	−0.3541 (3)	0.0407 (8)	
H30	−0.2360	0.8182	−0.3467	0.049*	
C31	−0.0594 (3)	0.78024 (15)	−0.2789 (3)	0.0338 (7)	
H31	−0.0972	0.7546	−0.2194	0.041*	

### Atomic displacement parameters (Å^2^)

**Table:**

	*U*^11^	*U*^22^	*U*^33^	*U*^12^	*U*^13^	*U*^23^
Pd1	0.02098 (11)	0.02433 (12)	0.02424 (11)	−0.00327 (8)	0.00494 (7)	0.00132 (8)
Cl2	0.0312 (4)	0.0368 (4)	0.0411 (4)	−0.0125 (3)	0.0065 (3)	0.0015 (3)
C3	0.0080 (8)	0.0087 (9)	0.0069 (9)	−0.0025 (7)	0.0057 (7)	−0.0015 (7)
P4	0.0200 (3)	0.0219 (3)	0.0212 (3)	−0.0011 (2)	0.0052 (2)	0.0004 (3)
C5	0.0223 (12)	0.0276 (14)	0.0243 (13)	−0.0017 (10)	0.0077 (10)	−0.0030 (11)
C6	0.0357 (15)	0.0345 (16)	0.0260 (14)	−0.0035 (13)	0.0034 (12)	0.0022 (12)
C7	0.0412 (17)	0.049 (2)	0.0231 (14)	−0.0068 (15)	0.0023 (12)	−0.0017 (13)
C8	0.0374 (16)	0.048 (2)	0.0296 (16)	−0.0045 (14)	0.0058 (13)	−0.0136 (14)
C9	0.0383 (17)	0.0307 (16)	0.0395 (17)	−0.0004 (13)	0.0090 (13)	−0.0085 (13)
C10	0.0305 (14)	0.0300 (15)	0.0269 (14)	0.0009 (12)	0.0079 (11)	−0.0007 (12)
C11	0.0251 (13)	0.0227 (13)	0.0215 (12)	−0.0010 (10)	0.0027 (10)	0.0004 (10)
C12	0.0251 (14)	0.0404 (18)	0.0379 (17)	0.0046 (12)	0.0080 (12)	0.0095 (14)
C13	0.0321 (16)	0.051 (2)	0.046 (2)	0.0076 (15)	−0.0019 (14)	0.0114 (16)
C14	0.0482 (19)	0.0397 (19)	0.0306 (16)	0.0012 (15)	−0.0047 (14)	0.0118 (14)
C15	0.0402 (17)	0.0419 (18)	0.0245 (14)	−0.0071 (14)	0.0053 (12)	0.0054 (13)
C16	0.0254 (13)	0.0342 (16)	0.0251 (13)	−0.0027 (11)	0.0030 (11)	0.0029 (12)
C17	0.0210 (12)	0.0263 (14)	0.0209 (12)	−0.0002 (10)	0.0038 (9)	0.0040 (10)
C18	0.0261 (13)	0.0257 (14)	0.0266 (14)	−0.0003 (11)	0.0026 (11)	0.0007 (11)
C19	0.0213 (12)	0.0338 (16)	0.0321 (15)	−0.0018 (11)	−0.0007 (11)	0.0060 (12)
C20	0.0214 (13)	0.0379 (17)	0.0335 (15)	0.0044 (12)	0.0051 (11)	0.0044 (13)
C21	0.0258 (13)	0.0339 (16)	0.0301 (14)	0.0046 (12)	0.0066 (11)	−0.0029 (12)
C22	0.0250 (13)	0.0261 (14)	0.0244 (13)	0.0022 (10)	0.0043 (10)	0.0017 (11)
C23	0.0294 (14)	0.0287 (15)	0.0269 (14)	0.0036 (12)	0.0072 (11)	−0.0040 (12)
N24	0.0276 (11)	0.0248 (12)	0.0283 (12)	−0.0005 (9)	0.0032 (9)	−0.0044 (10)
C25	0.0322 (15)	0.0324 (16)	0.0319 (15)	−0.0031 (12)	0.0053 (12)	−0.0130 (13)
C26	0.0294 (14)	0.0338 (16)	0.0265 (14)	−0.0056 (12)	0.0039 (11)	−0.0134 (12)
C27	0.0343 (16)	0.0465 (19)	0.0346 (16)	−0.0073 (14)	0.0087 (13)	−0.0080 (14)
C28	0.051 (2)	0.050 (2)	0.0379 (18)	−0.0067 (17)	0.0099 (15)	0.0018 (16)
C29	0.048 (2)	0.049 (2)	0.0426 (19)	0.0011 (17)	−0.0033 (16)	−0.0041 (17)
C30	0.0309 (16)	0.045 (2)	0.0463 (19)	−0.0017 (14)	0.0007 (14)	−0.0114 (15)
C31	0.0317 (15)	0.0350 (16)	0.0353 (16)	−0.0070 (13)	0.0067 (12)	−0.0092 (13)

### Geometric parameters (Å, °)

**Table:**

Pd1—C3	2.1233 (19)	C15—H15	0.9500
Pd1—N24	2.150 (2)	C16—H16	0.9500
Pd1—P4	2.1939 (7)	C17—C18	1.395 (4)
Pd1—Cl2	2.4035 (8)	C17—C22	1.410 (4)
C3—H3A	0.9800	C18—C19	1.394 (4)
C3—H3B	0.9800	C18—H18	0.9500
C3—H3C	0.9800	C19—C20	1.380 (4)
P4—C5	1.821 (3)	C19—H19	0.9500
P4—C17	1.825 (3)	C20—C21	1.387 (4)
P4—C11	1.825 (3)	C20—H20	0.9500
C5—C10	1.390 (4)	C21—C22	1.397 (4)
C5—C6	1.402 (4)	C21—H21	0.9500
C6—C7	1.391 (4)	C22—C23	1.474 (4)
C6—H6	0.9500	C23—N24	1.279 (4)
C7—C8	1.386 (5)	C23—H23	0.9500
C7—H7	0.9500	N24—C25	1.486 (4)
C8—C9	1.384 (5)	C25—C26	1.512 (4)
C8—H8	0.9500	C25—H25A	0.9900
C9—C10	1.392 (4)	C25—H25B	0.9900
C9—H9	0.9500	C26—C31	1.396 (4)
C10—H10	0.9500	C26—C27	1.399 (4)
C11—C12	1.393 (4)	C27—C28	1.384 (5)
C11—C16	1.396 (4)	C27—H27	0.9500
C12—C13	1.386 (4)	C28—C29	1.395 (5)
C12—H12	0.9500	C28—H28	0.9500
C13—C14	1.378 (5)	C29—C30	1.383 (5)
C13—H13	0.9500	C29—H29	0.9500
C14—C15	1.382 (5)	C30—C31	1.390 (5)
C14—H14	0.9500	C30—H30	0.9500
C15—C16	1.390 (4)	C31—H31	0.9500
			
C3—Pd1—N24	174.86 (9)	C15—C16—C11	120.1 (3)
C3—Pd1—P4	90.87 (6)	C15—C16—H16	120.0
N24—Pd1—P4	86.76 (7)	C11—C16—H16	120.0
C3—Pd1—Cl2	88.10 (6)	C18—C17—C22	118.7 (2)
N24—Pd1—Cl2	94.38 (7)	C18—C17—P4	120.8 (2)
P4—Pd1—Cl2	178.09 (3)	C22—C17—P4	120.4 (2)
Pd1—C3—H3A	109.5	C19—C18—C17	121.1 (3)
Pd1—C3—H3B	109.5	C19—C18—H18	119.5
H3A—C3—H3B	109.5	C17—C18—H18	119.5
Pd1—C3—H3C	109.5	C20—C19—C18	120.2 (3)
H3A—C3—H3C	109.5	C20—C19—H19	119.9
H3B—C3—H3C	109.5	C18—C19—H19	119.9
C5—P4—C17	102.71 (12)	C19—C20—C21	119.4 (3)
C5—P4—C11	106.07 (13)	C19—C20—H20	120.3
C17—P4—C11	104.43 (12)	C21—C20—H20	120.3
C5—P4—Pd1	121.09 (9)	C20—C21—C22	121.3 (3)
C17—P4—Pd1	110.79 (9)	C20—C21—H21	119.3
C11—P4—Pd1	110.33 (9)	C22—C21—H21	119.3
C10—C5—C6	119.3 (3)	C21—C22—C17	119.2 (3)
C10—C5—P4	122.0 (2)	C21—C22—C23	116.2 (2)
C6—C5—P4	118.6 (2)	C17—C22—C23	124.5 (2)
C7—C6—C5	120.2 (3)	N24—C23—C22	127.5 (3)
C7—C6—H6	119.9	N24—C23—H23	116.2
C5—C6—H6	119.9	C22—C23—H23	116.2
C8—C7—C6	119.9 (3)	C23—N24—C25	114.9 (2)
C8—C7—H7	120.1	C23—N24—Pd1	129.8 (2)
C6—C7—H7	120.1	C25—N24—Pd1	115.15 (17)
C9—C8—C7	120.3 (3)	N24—C25—C26	110.3 (2)
C9—C8—H8	119.9	N24—C25—H25A	109.6
C7—C8—H8	119.9	C26—C25—H25A	109.6
C8—C9—C10	120.1 (3)	N24—C25—H25B	109.6
C8—C9—H9	120.0	C26—C25—H25B	109.6
C10—C9—H9	120.0	H25A—C25—H25B	108.1
C5—C10—C9	120.3 (3)	C31—C26—C27	118.9 (3)
C5—C10—H10	119.9	C31—C26—C25	120.6 (3)
C9—C10—H10	119.9	C27—C26—C25	120.5 (3)
C12—C11—C16	119.1 (3)	C28—C27—C26	120.6 (3)
C12—C11—P4	118.9 (2)	C28—C27—H27	119.7
C16—C11—P4	121.9 (2)	C26—C27—H27	119.7
C13—C12—C11	120.2 (3)	C27—C28—C29	119.8 (3)
C13—C12—H12	119.9	C27—C28—H28	120.1
C11—C12—H12	119.9	C29—C28—H28	120.1
C14—C13—C12	120.4 (3)	C30—C29—C28	120.3 (4)
C14—C13—H13	119.8	C30—C29—H29	119.8
C12—C13—H13	119.8	C28—C29—H29	119.8
C13—C14—C15	120.0 (3)	C29—C30—C31	119.7 (3)
C13—C14—H14	120.0	C29—C30—H30	120.1
C15—C14—H14	120.0	C31—C30—H30	120.1
C14—C15—C16	120.2 (3)	C30—C31—C26	120.7 (3)
C14—C15—H15	119.9	C30—C31—H31	119.6
C16—C15—H15	119.9	C26—C31—H31	119.6
			
C3—Pd1—P4—C5	21.97 (12)	Pd1—P4—C17—C18	−144.6 (2)
N24—Pd1—P4—C5	−162.59 (13)	C5—P4—C17—C22	170.6 (2)
C3—Pd1—P4—C17	142.22 (11)	C11—P4—C17—C22	−78.9 (2)
N24—Pd1—P4—C17	−42.35 (11)	Pd1—P4—C17—C22	39.9 (2)
C3—Pd1—P4—C11	−102.64 (11)	C22—C17—C18—C19	−0.5 (4)
N24—Pd1—P4—C11	72.79 (11)	P4—C17—C18—C19	−176.0 (2)
C17—P4—C5—C10	101.3 (2)	C17—C18—C19—C20	1.0 (4)
C11—P4—C5—C10	−8.0 (3)	C18—C19—C20—C21	0.3 (4)
Pd1—P4—C5—C10	−134.6 (2)	C19—C20—C21—C22	−2.2 (5)
C17—P4—C5—C6	−74.6 (2)	C20—C21—C22—C17	2.7 (4)
C11—P4—C5—C6	176.1 (2)	C20—C21—C22—C23	180.0 (3)
Pd1—P4—C5—C6	49.5 (2)	C18—C17—C22—C21	−1.4 (4)
C10—C5—C6—C7	0.4 (4)	P4—C17—C22—C21	174.2 (2)
P4—C5—C6—C7	176.3 (2)	C18—C17—C22—C23	−178.4 (3)
C5—C6—C7—C8	−0.5 (5)	P4—C17—C22—C23	−2.8 (4)
C6—C7—C8—C9	0.3 (5)	C21—C22—C23—N24	161.0 (3)
C7—C8—C9—C10	0.0 (5)	C17—C22—C23—N24	−21.9 (5)
C6—C5—C10—C9	0.0 (4)	C22—C23—N24—C25	175.4 (3)
P4—C5—C10—C9	−175.9 (2)	C22—C23—N24—Pd1	−0.2 (5)
C8—C9—C10—C5	−0.1 (4)	P4—Pd1—N24—C23	31.1 (3)
C5—P4—C11—C12	−87.4 (3)	Cl2—Pd1—N24—C23	−147.4 (3)
C17—P4—C11—C12	164.5 (2)	P4—Pd1—N24—C25	−144.5 (2)
Pd1—P4—C11—C12	45.4 (3)	Cl2—Pd1—N24—C25	37.0 (2)
C5—P4—C11—C16	94.5 (2)	C23—N24—C25—C26	−102.6 (3)
C17—P4—C11—C16	−13.6 (3)	Pd1—N24—C25—C26	73.7 (3)
Pd1—P4—C11—C16	−132.7 (2)	N24—C25—C26—C31	−109.5 (3)
C16—C11—C12—C13	−0.9 (5)	N24—C25—C26—C27	72.0 (3)
P4—C11—C12—C13	−179.0 (3)	C31—C26—C27—C28	−0.9 (5)
C11—C12—C13—C14	0.8 (5)	C25—C26—C27—C28	177.6 (3)
C12—C13—C14—C15	−0.2 (6)	C26—C27—C28—C29	−0.1 (5)
C13—C14—C15—C16	−0.2 (5)	C27—C28—C29—C30	0.8 (5)
C14—C15—C16—C11	0.1 (5)	C28—C29—C30—C31	−0.3 (5)
C12—C11—C16—C15	0.4 (4)	C29—C30—C31—C26	−0.7 (5)
P4—C11—C16—C15	178.5 (2)	C27—C26—C31—C30	1.3 (4)
C5—P4—C17—C18	−14.0 (2)	C25—C26—C31—C30	−177.2 (3)
C11—P4—C17—C18	96.6 (2)		

## References

[bb1] Barbour, L. J. (2001). *J. Supramol. Chem* **1**, 189–191.

[bb2] Bruker (2007). *APEX2*, *SAINT-Plus* and *XPREP.* Bruker AXS Inc., Madison, Wisconsin, USA.

[bb3] Chiririwa, H., Meijboom, R. & Omondi, B. (2011). *Acta Cryst.* E**67**, m608–m609. 10.1107/S1600536811013936 PMC3089087 21754326

[bb4] Coleman, K. S., Green, M. L. H., Pascu, S. I., Rees, N. H., Cowley, A. R. & Rees, L. H. (2001). *J. Chem. Soc. Dalton Trans* pp. 3384–3395.

[bb5] Ghilardi, C. A., Midollini, S., Moneti, S., Orlandini, A. & Scapacci, G. (1992). *J. Chem. Soc. Dalton Trans.* pp. 3371–3376.

[bb6] Sanchez, G., Momblona, F., Perez, J. & Lopez, G. (2001). *Transition Met. Chem.* **26**, 100–104.

[bb7] Sanchez, G., Serrano, J. L., Moral, M. A., Perez, J., Molins, E. & Lopez, G. (1999). *Polyhedron*, **18**, 3057–3064.

[bb8] Sanchez, G., Serrano, J. L., Ruiz, F. & Lopez, G. (1998). *J. Fluorine Chem.* **91**, 165–169.

[bb9] Sheldrick, G. M. (1997). *SADABS* University of Göttingen, Germany.

[bb10] Sheldrick, G. M. (2008). *Acta Cryst.* A**64**, 112–122. 10.1107/S0108767307043930 18156677

